# Supplementary material for: Poor compliance with school food environment guidelines in elementary schools in Northwest Mexico: A cross-sectional study
Source: PLoS One. 2021 Nov 11;16(11):e0259720. doi: 10.1371/journal.pone.0259720 (PMC8584694; doi:10.1371/journal.pone.0259720)
Supplement: S1 File — General data, Interview with school authorities, School canteen instrument, Breakfast menu instrument, Evaluation of the physical environment, Non-participation survey, and Checklist. (PDF) [file pone.0259720.s002.pdf]

S1 File. Data collection instruments (Spanish versions) – 1. General data, 2. Interview with school authorities, 3. School canteen instrument, 4. Breakfast menu instrument, 5. Evaluation of the physical environment, 6. Non-participation survey, and 7. Checklist.

Table 1. General Data

FOLIO: _________

UNIVERSIDAD DE SONORA
DEPARTAMENTO EN CS. QUÍMICO BIOLÓGICAS Y DE LA SALUD
MAESTRÍA EN CIENCIAS DE LA SALUD

DATOS DE LA ESCUELA

NOMBRE:
CLAVE:
DIRECCIÓN:
TURNO:
PÚBLICA ☐ PRIVADA ☐
NÚMERO DE ALUMNOS:

DATOS DE CONTACTO

NOMBRE:
PUESTO/RESPONSABILIDAD:
CORREO ELECTRÓNICO:
TELÉFONO:

DATOS DE LA VISITA

FECHA:			
HORA DE LLEGADA:			
TIEMPO EN EL QUE SE RECOPILARON LOS DATOS:	☐ ANTES DEL RECREO	☐ DURANTE EL RECREO	☐ DESPUÉS DEL RECREO
NOMBRE DEL EVALUADOR/ES:			

Table 2. Interview with School Authorities

FOLIO: _________

Entrevista con Directivos

Reactivo	Respuesta
**1.** ¿Actualmente su escuela participa en algún programa de desayunos o comidas escolares?	☐ No (0) * ☐ Si (1)<br>*Si la respuesta es "No", pasar a pregunta 4, Si la respuesta es "Si"…*
¿El programa de alimentos es dirigido por el DIF (Sist. de Desarrollo Integral de la Familia)?	☐ No (0) ☐ Si (1) ☐ Otra(2):______________________
**2.** Para la elaboración de alimentos, ¿La escuela cuenta con cocina propia?	☐ No (0) ☐ Si (1)<br>*Si la respuesta es "No" pasar a la pregunta siguiente, si la respuesta es "Si",…*
¿La cocina está dentro de la escuela?	☐ No (0) ☐ Si (1)
**3.** ¿Tiene a la mano alguna copia del menú de desayunos o comidas escolares?	☐ No (0) ☐ Si (1)<br>*Si la respuesta es "Si", solicitar copia y responda:* ***¿Existe copia?***__________
* **4.** ¿Hay algún lugar dentro o fuera de la escuela donde los alumnos compren normalmente sus alimentos?	☐ No (0) ☐ Si (1)<br>*Si la respuesta es "No", pasar a la pregunta siguiente, si la respuesta es "Si",…*
A continuación voy a mencionarle algunos lugares donde se venden alimentos, ¿Podría indicarme en cuáles de estos los alumnos normalmente compran su comida?	☐ Comedor escolar ☐ Tienda escolar/ Cooperativa ☐ Maquina expendedora ☐ Vendedores ambulantes<br>☐ Los maestros o administrativos ☐ Otro/s:______________________
**5.** Para la recaudación de fondos ¿La escuela ha utilizado la venta de alimentos o bebidas? Ej. Barras de chocolate.	☐ No (0) ☐ Si (1)<br>*Si la respuesta es "No", pasar a la pregunta siguiente, si la respuesta es "Si",…*
A continuación voy a mencionarle algunos alimentos que normalmente se utilizan para esta actividad, ¿Podría indicarme cuál o cuáles de estos se han utilizado?	☐ Barras de chocolate ☐ Pasteles, pays o galletas ☐ Frituras de papa o maíz ☐ Alimentos preparados<br>☐ Otro/s:______________________

**6.** ¿Sabe si existe algún tipo de control hacia los vendedores ambulantes que se colocan fuera de la escuela?	☐ No (0)	☐ Si (1)	☐ No sabe (2)	
	Si la respuesta es "No" o "No sabe", pasar a la pregunta siguiente, si la respuesta es "Si",…			
De las siguientes opciones, ¿Podría indicarme de dónde proviene este reglamento o regulación?	☐ Políticas internas, es decir, elaboradas por la propia escuela (0)	☐ Políticas externas, es decir, promovidas por instituciones de gobierno (1)	☐ Otro(2):______ ____________ ____________	☐ No sabe (3)

**7.** ¿Su escuela cuenta con bebederos o enfriadores para el agua?	☐ No (0)	☐ Si (1)		
¿Cuántas de estas fuentes de agua pueden utilizar los alumnos?	☐ Solo un bebedero (0)	☐ Dos a tres bebederos (1)	☐ Cuatro o cinco bebederos (2)	☐ Seis bebederos o más (3)

**8.** ¿El agua de estas fuentes está disponible durante todo el año?	☐ No (0)	☐ Si (1)	☐ No sabe (2)

**9.** ¿Considera que las fuentes de agua disponibles son suficientes para los alumnos de esta escuela?	☐ No (0)	☐ Si (1)	☐ No sabe (2)	
	Si la respuesta es "Si", pasar a la pregunta siguiente, si la respuesta es "No",…			
A continuación voy a mencionarle diferentes situaciones sobre el agua, ¿Podría indicarme cuál o cuáles suceden en esta escuela?	☐ Los alumnos tienen que comprar el agua	☐ No hay bebederos al alcance de los alumnos	☐ Los bebederos están sucios o no funcionan bien	☐ Otra:________ ____________ ____________

**10.** ¿El reglamento escolar incluye políticas relacionadas con comida chatarra o bebidas azucaradas?	☐ No (0)	☐ Si (1)	☐ No sabe (2)	
	Si la respuesta es "No" o "No sabe", pasar a la pregunta 12, si la respuesta es "Si",…			
A continuación voy a mencionarle algunas situaciones o eventos especiales, indique en cuál o cuáles se permite que los alumnos consuman estos alimentos (comida chatarra y bebidas azucaradas).	☐ Festivales o kermeses	☐ Fiestas de cumpleaños	☐ Días festivos o especiales	☐ Todos los días
	☐ Otro/s:____ ____ ____ ____ ____			

11. ¿El reglamento de la escuela prohibe la venta o preparación de ciertos alimento y bebidas en el comedor, tienda o cualquier otro lugar de la escuela al que tengan acceso los alumnos?

☐ No (0) ☐ Si (1) ☐ No sabe (2)

Si la respuesta es "No" o "No sabe", pasar a la pregunta siguiente, si la respuesta es "Si",…

¿Estas reglas varian los diferentes días de la semana?

☐ No (0) ☐ Si (1) ☐ No sabe (2)

Si la respuesta es "Si", especifique qué días y cuáles son las diferencias:

____

____

____

____

____

____

____

12. ¿Algún porcentaje de las ventas de alimentos se destina a la escuela?

☐ No (0) ☐ Si (1)

Si la respuesta es "No", pasar a la pregunta siguiente, si la respuesta es "Si",…

¿Podría decirme que tipo de necesidades se cubren con este apoyo económico?

____

____

____

____

____

13. ¿La escuela ha recibido visitas de autoridades que verifiquen la elaboración, venta y distribución de alimentos en la escuela?

☐ No (0) ☐ Si (1)

Si la respuesta es "No", pasar a la pregunta siguiente, si la respuesta es "Si",…

Indique por favor qué grupo o dependencia visitó la escuela

☐ Secretaría de Salud ☐ Secretaría de Educación ☐ Otra: ______________________________

____

14. En México existe una Norma que se llama "Acuerdo mediante el cual se establecen los lineamientos generales para el expendio y distribución de alimentos y bebidas preparados y procesados en las escuelas del Sistema Educativo Nacional" ¿Ha recibido información relacionada con este Acuerdo?

☐ No (0) ☐ Si (1)

Si la respuesta es "No", pasar a la pregunta 16 y explicar con apoyo del resumen que está a continuación, si la respuesta es "Si"...

Resumen: El Acuerdo se creó el año 2010, su versión más actual es del año 2014. Incluye las características de los alimentos y bebidas que pueden o no estar disponibles en las escuelas, ya sea en el menú del comedor o lo que se vende en las cafeterías o tienditas. Recomienda y prohibe ciertos alimentos de acuerdo a su calidad nutricional y para algunos de ellos especifica el tamaño límite de la porción por envase.

A continuación voy a mencionarle diferentes personas e instituciones, indique cuál o cuáles de ellas le proporcionaron información respecto al Acuerdo.	☐ Compañeros de trabajo ☐ Medios de comunicación ☐ Autoridades de gobierno ☐ Jefe o Superior<br>☐ Otro/s:______________________________<br>______________________________
**15.** ¿Ha recibido capacitación formal relacionada con el acuerdo anteriormente mencionado?	☐ No (0) ☐ Si (1)<br>*Si la respuesta es "No", pasar a la siguiente pregunta, si la respuesta es "Si"…*
A continuación, voy a mencionarle diferentes personas e instituciones, indique cuál o cuáles le proporcionaron capacitación relacionada al Acuerdo.	☐ Secretaría de Educación ☐ Secretaría de Salud ☐ Compañeros de trabajo ☐ Jefe o Superior<br>☐ Otro/s:______________________________<br>______________________________
**16.** De los siguientes grupos o personas, indique todos aquellos que considera pueden dificultar la aplicación del Acuerdo en esta escuela.	☐ Padres de familia ☐ Maestros de la escuela ☐ Autoridades de la escuela ☐ Autoridades e instituciones externas<br>☐ Proveedores y vendedores ☐ Los alumnos ☐ Otro/s:______<br>__________<br>__________<br>*Si no se selecciona alguna opción, pasar a la pregunta 19..*
**17.** ¿De que forma cree que pueden dificultar la aplicación del Acuerdo los grupos o personas que indicó anteriormente?	☐ Falta de apoyo económico ☐ Falta de interés ☐ Falta de recurso humano ☐ No conocen el Acuerdo<br>☐ No están dispuestos a dar o recibir capacitación ☐ Otro/s:______________________________<br>______________________________<br>______________________________

**18.** ¿Qué cree que se puede hacer para mejorar lo anterior?	______________________________			
**19.** A continuación voy a mencionarle algunas opciones, indique las que cree que pueden favorecer a la aplicación del Acuerdo.	☐ Padres de familia<br>☐ Proveedores y vendedores<br>*Si no se selecciona alguna opción, pasar a la pregunta 21..*	☐ Maestros de la escuela<br>☐ Los alumnos	☐ Autoridades de la escuela<br>☐ Otro/s:______	☐ Autoridades e instituciones externas
**20.** ¿De qué manera cree que pueden favorecer ayudar a la aplicación del Acuerdo en la escuela los grupos y personas que indicó anteriormente?	☐ Brindando apoyo<br>☐ Estando dispuestos a dar o recibir capacitación	☐ Mostrando interés<br>☐ Otro/s:______	☐ Apoyando con recurso humano	☐ Difundiendo información
**21.** Hasta antes de esta entrevista, ¿Había escuchado sobre este Acuerdo?	☐ No (0)<br>*Si la respuesta es "No", pasar a pregunta 27.*	☐ Si (1)		
**22.** ¿Cuenta con una copia impresa o digital del Acuerdo?	☐ No (0)<br>*Si la respuesta es "Si", solicitar copia y responda:* ***¿Existe copia?________***	☐ Si (1)		
**23.** ¿Cree que la escuela está implementando lo contenido en el Acuerdo?	☐ No (0)<br>*Si la respuesta es "No", pasar a la pregunta siguiente, si la respuesta es "Si"…*	☐ Sí, apenas estamos iniciando trabajos para implementar (1)	☐ Sí, llevamos tiempo trabajando en su implementación [illegible]	☐ Ya está completamente implementado o casi por completo (3)

¿Desde qué fecha se han realizado trabajos para la implementación del Acuerdo?	Día_______ Mes__________________ Año____________		
**24.** ¿Los maestros de la escuela tienen acceso a una copia impresa o digital del Acuerdo?	☐ No (0)	☐ Si (1)	☐ No sabe (2)
**25.** ¿Las personas responsables de la tienda o cafetería escolar tienen acceso a una copia impresa o digital del Acuerdo?	☐ No (0)	☐ Si (1)	☐ No sabe (2)
**26.** ¿Los padres de familia reciben algún tipo de información respecto al Acuerdo?	☐ No (0)	☐ Si (1)	☐ No sabe (2)
**27.** ¿Existe algún comité que regule la venta y distribución de alimentos y bebidas dentro de la escuela?	☐ No (0)	☐ Si (1)	☐ No sabe (2)
**28.** Dentro de la escuela, ¿Existe algún tipo de material de divulgación relacionado con el Acuerdo que hablamos?	☐ No (0)	☐ Si (1)	☐ No sabe (2)

Comentarios:____

____

____

Table 3. School Canteen Instrument

FOLIO: _________

Alimentos Disponibles en la Tienda Escolar

Antes de llenar el cuestionario responda lo siguiente:

Este instrumento fue aplicado a: Tienda/Cooperativa ☐ Máquina expendedora ☐ Otro:____________________ ☐

Alimento/Bebida	Categoría		
	Verde	Ámbar	Rojo
**Agua**	Agua potable ☐		
**Frutas y verduras**	Fruta y verdura fresca picada o entera ☐		
**Jugo natural de frutas o verduras**		En jugo (100% natural) sin azúcar añadida ☐	En jugo (100% natural) con azúcar añadida ☐
**Cereales integrales (amaranto, avena o granola)**	Sin azúcar añadida ☐		Con azúcar añadida ☐
**Semillas y nueces**	Sin sal añadida ☐		Con sal añadida ☐
**Leguminosas secas (cacachuates, habas)**	Sin sal añadida ☐		Con sal añadida ☐
**Leche**	Entera, semi o descremada sin azúcar añadida ☐	Entera, semi o descremada con edulcurantes artificiales ☐	Entera, semi o descremada con azúcar añadida ☐
**Bebidas a base de leche**	Entera, semi o descremada sin azúcar añadida ☐	Entera, semi o descremada con edulcurantes artificiales ☐	Entera, semi o descremada con azúcar añadida ☐
**Bebidas deportivas o energéticas**			Cualquier presentación ☐
**Bebidas de soya**	Sin azúcar añadida ☐	Con edulcurantes artificiales ☐	Con azúcar añadida ☐
**Quesos de refrigerio**	Bajos en grasa y sodio ☐		Altos en grasas y/o sodio ☐
**Néctares y jugos procesados**			Cualquier presentación ☐
**Tés, refrescos y otras bebidas azucaradas**			Cualquier presentación ☐
**Botanas (papas fritas y otros alimentos procesados salados)**			Cualquier presentación ☐
**Galletas, pastelitos, dulces y postres**			Cualquier presentación ☐

Alimentos preparados			
**Alimento/Bebida**	**Categoría**		
	**Verde**	**Ámbar**	**Rojo**
**Sándwich**	Pan integral y con verdura ☐		Pan blanco y sin vegetales ☐
**Quesadillas**	Tortilla de maíz con queso ☐		Tortilla de harina con queso ☐
**Tacos y burritos**	Con tortilla de maíz ☐		Con tortilla de harina ☐
**Sopa**	Casera y con vegetales ☐		Sopa instantánea ☐
**Bolis, nieve o paletas de hielo**			Cualquier presentación ☐
**Duros (fritura de maíz)**			Cualquier presentación ☐
**Pizza**			Cualquier presentación ☐
**Molletes**			Cualquier presentación ☐
**Pepihuates**			Cualquier presentación ☐
**Tamales**			Cualquier presentación ☐
**Torta**			Cualquier presentación ☐
**Otros:**			

100% (o cerca del 100%) de los artículos disponibles en la tienda escolar están clasificados en verde o ámbar ☐ SI ☐ NO

Al menos **50%** de los artículos disponibles en la tienda escolar están clasificados como verde o ámbar ☐ SI ☐ NO

Table 4. Breakfast Menu Instrument

FOLIO: __________

Contenido del Menú de Desayunos/Comidas Escolares

Alimentos utilizados para la elaboración de platillos		
**Verde**	**Ámbar**	**Rojo**
Verduras ☐		
Cereales integrales o de grano entero ☐		Cereales que no son integrales o de grano entero (tortilla de harina, pasta, etc.) ☐
Leguminosas o Productos de origen animal (carne de res, pollo, cerdo, etc.) ☐		Embutidos y otros productos de origen animal con alto contenido de sodio ☐
Frutas ☐		
Agua simple ☐		
Atoles a base de cereales integrales (avena, amaranto, cebada, arroz) ☐		Atoles a base de cereales NO integrales (fécula de maíz, galleta, etc.) ☐
		Mantequilla o Crema ☐
Fruta deshidratada ☐		
Semillas y nueces ☐		

Otros alimentos agregados al menú		
**Verde**	**Ámbar**	**Rojo**
	Jugos de fruta/verduras 100% natural y sin azúcar añadida ☐	Jugos de fruta/verduras 100% natural con azúcar añadida ☐
		Néctares y jugos procesados ☐
		Néctares y jugos procesados con edulcorantes ☐
		Tés, refrescos y otras bebidas azucaradas no lácteas ☐
		Tés, refrescos y otras bebidas azucaradas no lácteas con edulcorantes ☐
Quesos para refrigerio bajos en grasas y sodio ☐		Quesos para refrigerio altos en grasas y/o sodio ☐
Leche entera, semi o descremada sin azúcar añadida ☐	Leche entera, semi o descremada con edulcurantes artificiales ☐	Leche entera, semi o descremada con azúcar añadida ☐
Bebidas de soya sin azúcar añadida ☐	Bebidas de soya con edulcorantes artificiales ☐	Bebidas de soya con azúcar añadida ☐
		Botanas (papas fritas y otros alimentos procesados salados) ☐
		Galletas, pastelitos, confites y postres ☐
Otros: ☐	☐	☐

100% (o cerca del 100%) de los alimentos incluidos en el menú escolar están clasificados en verde o ámbar → SI ☐ NO ☐

Al menos **50%** de los alimentos incluidos en el menú escolar están clasificados como verde o ámbar → SI ☐ NO ☐

Table 5. Evaluation of the physical environment

Evaluación Estructural en Base a la Observación

Reactivo	Respuesta		
**1.** ¿Se observa publicidad dirigida a bebidas y/o alimentos procesados en la escuela?	☐ No (0)	☐ Si (1)	
**2.** ¿Se observan publicaciones referentes a lo que indica el Acuerdo dentro de la escuela?	☐ No (0)	☐ Si (1)	
**3.** ¿Se observan vendedores ambulantes al exterior de la escuela?<br>*Considerar solo aquellos puestos/vendedores ambulantes en la misma calle frente a la escuela*	☐ No (0)<br>*Si la respuesta es "No", pasar a la pregunta 4, si la respuesta es "Si"...*	☐ Si (1)	
Número de puestos/vendedores ambulantes al exterior de la escuela:	____________________		
Categoría en la que se encuentran la mayoría de los alimentos en los puestos ambulantes al exterior de la escuela:	☐ Rojo *SSBs y HSFAS* (0)	☐ Ámbar *Jugos de fruta s/azúcar* (1)	☐ Verde *Vegetales, Cereales enteros* (2)
**4.** ¿Existen bebederos o algún tipo de acceso al agua potable gratuita en la escuela?	☐ No (0)<br>*Si la respuesta es "No", omita el resto de las preguntas, si la respuesta es "Si",…*	☐ Si (1)	
Especifique cuántos bebederos o fuentes de agua potable observó:	____________________		
¿Todos los bebederos o fuentes son funcionales?	☐ No (0)<br>*Si la respuesta es "No", **Especifique cuántos son funcionales:** _______*	☐ Si (1)	
¿Todos los bebederos o fuentes están limpios?	☐ No (0)<br>*Si la respuesta es "No", **Especifique cuántos están limpios:** _______*	☐ Si (1)	

FOTOGRAFÍAS INCLUIDAS EN EL REGISTRO:

2 Fotografías del exterior (vendedores ambulantes)	☐ Si	☐ No	Comentarios:
2 Fotografías de la tienda escolar	☐ Si	☐ No	
2 Fotografías de publicidad (SSBs o HSFAS)	☐ Si	☐ No	
2 Fotografías de publicaciones que promuevan hábitos saludables	☐ Si	☐ No	

Table 6. Non-participation Survey

FOLIO: _________

Instrucciones: En el dado caso que las autoridades responsables de la escuela rechacen su participación en el estudio, solicitar de la manera más amable la siguiente información:

ENCUESTA DE NO PARTICIPACIÓN

"Gracias por su tiempo, antes de irnos quisieramos que nos ayude a responder 3 preguntas rápidas, nos sería de mucha ayuda"

1. ¿El reglamento escolar incluye políticas que regulen el consumo de comida chatarra y bebidas azucaradas?

☐ No (0) ☐ Si (1) ☐ No sabe (2)

2. ¿Considera que las fuentes de agua disponibles son suficientes para los alumnos de esta escuela?

☐ No (0) ☐ Si (1) ☐ No sabe (2)

3. En México existe una Norma que se llama "Acuerdo mediante el cual se establecen los lineamientos generales para el expendio y distribución de alimentos y bebidas preparados y procesados en las escuelas del Sistema Educativo Nacional" ¿Ha recibido información relacionada con este Acuerdo?

☐ No (0) ☐ Si (1)

Fig 1. Checklist

Folio: ___________

CHECK LIST DE VISITA

PASO 1 (Antes de la visita):
- ☐ Materiales (7)
- ☐ Hoja de identificación (sección 1)
- ☐ Asignar folios
- ☐ Portar gafete

PASO 2 (Al llegar):
- ☐ Hoja de identificación (sección 3)
- ☐ Entrar a la escuela (buscar director)
- ☐ Acceso negado

PASO 3 (Al entrar):
- ☐ Presentarse
- ☐ Beneficios del estudio
- ☐ Consentimiento informado
- ☐ Firma o Encuesta de no participación
- ☐ Hoja de identificación (sección 2)

PASO 4 (Entrevista):
- ☐ Anonimato
- ☐ Explicar dinámica

PASO 5 (Menú escolar):
- ☐ Formato de Menú
- ☐ Canalizar con responsable
- ☐ Vía correo (anotar)

PASO 6 (Tiendita escolar):
- ☐ Ir a la/s tiendita/s
- ☐ Fotos de tiendita
- ☐ Formato de tienditas
- ☐ ¿Existe otro lugar?

PASO 7 (Evaluación estructural):
- ☐ Evaluación estructural
- ☐ Fotos de publicidad y del Acuerdo

PASO 8 (Despedida):
- ☐ Agradecimiento
- ☐ Envío de informe
- ☐ Formatos completos
- ☐ Salir
- ☐ Fotos de vendedores ambulantes
