# Supplementary material for: Poor compliance with school food environment guidelines in elementary schools in Northwest Mexico: A cross-sectional study
Source: PLoS One. 2021 Nov 11;16(11):e0259720. doi: 10.1371/journal.pone.0259720 (PMC8584694; doi:10.1371/journal.pone.0259720)
Supplement: S2 File — General data, Interview with school authorities, School canteen instrument, Breakfast menu instrument, Evaluation of the physical environment, Non-participation survey, and Checklist. (PDF) [file pone.0259720.s003.pdf]

S2 File. Data collection instruments (English versions)– General data, Interview with school authorities, School canteen instrument, Breakfast menu instrument, Evaluation of the physical environment, Non-participation survey, and Checklist.

Table 1. General Data

SCHOOL NUMBER: _________

UNIVERSITY OF SONORA
DEPARTMENT OF CHEMICAL-BIOLOGICAL AND HEALTH SCIENCES
MASTER IN HEALTH SCIENCES

SCHOOL INFORMATION

NAME:
IDENTIFICATION NUMBER:
ADDRESS:
SHIFT (morning or afternoon):
PUBLIC ☐ PRIVATE ☐
NUMBER OF STUDENTS:

CONTACT INFORMATION

NAME:
POSITION/TITLE:
EMAIL:
TELEPHONE NUMBER:

INFORMATION ABOUT THE VISIT

DATE:
TIME OF ARRIVAL:
TIME OF DATA COLLECTION: ☐ BEFORE PLAYTIME/RECESS ☐ DURING PLAYTIME/RECESS ☐ AFTER PLAYTIME/RECESS
NAMES OF DATA COLLECTORS:

Table 2. Interview with School Authorities

SCHOOL NUMBER: _________

School Authorities Interview

Question	Answer
**1.** Does your school currently participate in any school breakfast or lunch program?	☐ No (0) * ☐ Yes (1)<br>*If the answer is "no", continue with question 4. If the answer is "yes"...*
Is the program directed by DIF (National system of integral family development)?	☐ No (0) ☐ Yes (1) ☐ Other(2):_______
**2.** Does your school have its own kitchen for food preparation, ?	☐ No (0) ☐ Yes (1)<br>*If the answer is "no", continue with the next question. If the answer is "yes"...*
Is the kitchen inside the school?	☐ No (0) ☐ Yes (1)
**3.** Do you have a copy of the breakfast or lunch menu at hand?	☐ No (0) ☐ Yes (1)<br>*If the answer is "yes", ask to see the copy and answer: Did he/she provide the copy? ___________*
**4.** Is there at least one place inside or outside the school where children regularly buy food or beverages?	☐ No (0) ☐ Yes (1)<br>*If the answer is "no", continue with the next question. If the answer is "yes"…*
Next, I will mention some places that usually sell food. Could you tell me in which of them your students usually buy their food? (Note all that apply.)	☐ The school dining room ☐ The school canteen ☐ A vending machine ☐ From mobile food vendors<br>☐ From teachers or administrative staff ☐ Other/s:_______
**5.** Has your school sold food or drinks for fundraising, e.g. chocolate bars?	☐ No (0) ☐ Yes (1)<br>*If the answer is "no", continue with the next question. If the answer is "yes"…*
Next, I will mention different foods that are usually sold for fundraising. Could you tell me which of them your school has used? (Note all that apply.)	☐ Chocolate bars ☐ Cakes, pies or cookies ☐ Potato or corn chips ☐ Prepared food (i.e. Mexican food*)<br>☐ Other/s:_______

**6.** Do you know if there is some kind of control of mobile food vendors outside of the school?	☐ No (0)	☐ Yes (1)	☐ I don't know (2)	
	If the answer is "no" or "I don't know", continue with the next question. If the answer is "yes"…			
From the following options, could you incidate where this rule or regulation comes from?	☐ From the school's own policies (0)	☐ From a government institution (1)	☐ Other(2):_____ ____________ ____________	☐ Don't know (3)
**7.** Does your school have water fountains or water coolers?	☐ No (0)	☐ Yes (1)		
How many of these water fountains are available for students?	☐ Just one (0)	☐ Two or three (1)	☐ Four or five (2)	☐ Six or more (3)
**8.** Is the water from these sources available all year?	☐ No (0)	☐ Yes (1)	☐ I don't know (2)	
**9.** Do you consider that your school has enough water fountains for the students?	☐ No (0)	☐ Yes (1)	☐ I don't know (2)	
	If the answer is "no" or "I don't know", continue with the next question. If the answer is "yes"…			
Next, I will mention different situations about water. Could you tell me which of these situations occurs in your school?	☐ The students have to buy bottled water	☐ The water fountains are not within reach of the students	☐ The water fountains are dirty or do not function very well	☐ Other:________ ____________ ____________
**10.** Does the school have policies related to junk food or sugar sweetened beverages?	☐ No (0)	☐ Yes (1)	☐ I don't know (2)	
	If the answer is "no" or "I don't know", continue with the question 12. If the answer is "yes"…			
Next, I will mention some events or special occasions. Please let me know in which of them your school allows the students to consume junk food or sugar sweetened beverages. (Note all that apply.)	☐ Festivals or fetes	☐ Birthday parties	☐ Festive or special days	☐ Every day
	☐ Other/s:____			

11. Does your school have policies that prohibit the sale or preparation of certain food or beverages in the school canteen or any other place in the school to which the students have access?

☐ No (0) ☐ Yes (1) ☐ I don't know (2)

If the answer is "no" or "I don't know", continue with the next question. If the answer is "yes"…

Do these policies change on different days of the week?

☐ No (0) ☐ Yes (1) ☐ I don't know (2)

If the answer is "yes", please specify which days and what are the differences:

____

____

____

____

____

____

____

12. Does your school receive a percentage of the income from the sale of foods and beverages in the school canteen?

☐ No (0) ☐ Yes (1)

If the answer is "no", continue with the next question. If the answer is "yes"…

Could you tell me what kind of necessities are covered with this money?

____

____

____

____

____

13. Has your school received visits from an external authority to verify the preparation, sale and distribution of foods and beverages in the school?

☐ No (0) ☐ Yes (1)

If the answer is "no", continue with the next question. If the answer is "yes"…

Please indicate which group or government department visited the school.

☐ The Ministry of Health ☐ The Ministry of Education ☐ Other:______________________

____

14. In Mexico there is a guideline called "Agreement that establishes the general guidelines for the sale and distribution of prepared and processed foods and beverages in the schools of the National Education System." Have your received information about this Agreement?

☐ No (0) ☐ Yes (1)

If the answer is "no", continue with question 16 and explain what it is with the support of the summary below, if the answer is "yes"...

Summary: The Agreement was created in 2010, its current version is from 2014. It specifies the characteristics of the food and beverages that are allowed to be sold or distributed in all schools. The Agreement recommends and prohibits certain foods according to their nutritional quality and for some of them the serving size limit per container/packet is specified.

Next, I will mention different people and institutions. Please indicate which of them provided you with information about the Agreement. (Note all that apply.)

☐ Co-workers ☐ Communication media ☐ Government authorities ☐ Boss or superior

☐ Others:____

____

15. Have you received formal training related to the previously mentioned Agreement?

☐ No (0) ☐ Yes (1)

If the answer is "no", continue with the next question. If the answer is "yes"…

Next, I will mention different people and institutions. Please tell me which of them provided you with formal training related to the Agreement. (Note all that apply.)

☐ Ministry of Education ☐ Ministry of Health ☐ Co-workers ☐ Boss or superior

☐ Other/s:____

____

16. Please indicate which of the following groups or people you consider could (or do) make the application of the Agreement in your school difficult. (Note all that apply.)

☐ Parents and guardians ☐ Teachers ☐ School authorities ☐ External institutions and authorities

☐ Food vendors ☐ Students ☐ Other/s:______

____

____

If no option is selected, go to question 19.

17. For what reason do you think the groups or people you mentioned above could (or do) make the application of the Agreement difficult?

☐ Lack of financial support ☐ Lack of interest ☐ Lack of human resources ☐ They don't know the Agreement

☐ They are not interested in giving or receiving ☐ Other/s:______________________________

____

____

**18.** What do you think could be done to improve the above?	______________________________<br>______________________________<br>______________________________<br>______________________________<br>______________________________<br>______________________________<br>______________________________<br>______________________________<br>______________________________
**19.** From the following options, indicate those that you believe could (or do) facilitate the application of the Agreement in your school. (Note all that apply.)	☐ Parents and guardians ☐ Teachers ☐ School authorities ☐ External institutions and authorities<br>☐ Food vendors ☐ Students ☐ Other/s:______ ____________ ____________ ____________<br>*If no option is selected, go to question 21*
**20.** In what way do you think the people or groups you mentioned above could (or do) facilitate the application of the Agreement in your school?	☐ Providing financial support ☐ Showing interest ☐ Providing human resources ☐ Spreading information<br>☐ Being available to give or receive training ☐ Other/s:______________________________ ______________________________ ______________________________
**21.** Had you heard about the Agreement prior to this interview?	☐ No (0) ☐ Yes (1)<br>*If the answer is "no", continue with question 27.*
**22.** Do you have a printed or digital copy of the Agreement?	☐ No (0) ☐ Yes (1)<br>*If the answer is "yes", ask to see the copy,* ***Does the copy exist?*** __________
**23.** Do you think your school is implementing the contents of the Agreement?	☐ No (0) ☐ Yes, we have begun work to implement it (1) ☐ Yes, we began some time ago but are still working to complete it (2) ☐ Yes, it is completely, or almost completely, implemented (3)<br>*If the answer is "no", continue with the next question. If the answer is "yes"…*

From which date did your school begin working to implement the Agreement?	Day_______	Month__________________	Year_____________
**24.** Do the school teachers have access to a printed or digital copy of the Agreement?	☐ No (0)	☐ Yes (1)	☐ I don't know (2)
**25.** Do the people responsible of the school canteen have access to a printed or digital copy of the Agreement?	☐ No (0)	☐ Yes (1)	☐ I don't know (2)
**26.** Do the parents and guardians receive information about the Agreement?	☐ No (0)	☐ Yes (1)	☐ I don't know (2)
**27.** Is there a committee that regulates the sale and distribution of food and beverages in the school?	☐ No (0)	☐ Yes (1)	☐ I don't know (2)
**28.** Inside the school, is there any kind of promotional material related to the Agreement?	☐ No (0)	☐ Yes (1)	☐ I don't know (2)

Comments:____
____
____

Table 3. School Canteen Instrument

SCHOOL NUMBER: _________

Foods and Beverages Available in the School Canteen

Before completing the checklist, answer the following:

This instrument was applied to a: School canteen ☐ Vending machine ☐ Other:____________________ ☐

Food/Beverage	Category		
	Green	Amber	Red
**Bottled water**	Any presentation ☐		
**Fresh fruit and vegetables**	Any presentation ☐		
**Natural fruit juices (100% juice)**		Without added sugar ☐	With added sugar ☐
**Whole grain cereals (amaranth, oats or granola)**	Without added sugar ☐		With added sugar ☐
**Seeds and nuts**	Without added salt ☐		With added salt ☐
**Dried legumes (e.g. chickpeas, broad, beans)**	Without added salt ☐		With added salt ☐
**Whole, low-fat or skim milk**	Without added sugar ☐	With artificial sweeteners ☐	With added sugar ☐
**Milk-based beverages (e.g. milkshakes, hot chocolate)**	Without added sugar ☐	With artificial sweeteners ☐	With added sugar ☐
**Sports or energy drinks**			Any sport or energy drink ☐
**Soy drinks**	Without added sugar ☐	With artificial sweeteners ☐	With added sugar ☐
**Snack cheeses**	Low in fat and sodium ☐		High in fat or sodium ☐
**Processed juices and nectars**			Any presentation ☐
**Iced tea, sodas and other sugar sweetened beverages**			Any presentation ☐
**Snacks (potato chips and other salty processed foods)**			Any presentation ☐
**Cookies, cakes, candies and sweets**			Any presentation ☐

Prepared foods			
**Food/Beverage**	**Category**		
	**Green**	**Amber**	**Red**
**Sandwiches**	Whole grain sandwiches with vegetables ☐		White bread sandwiches without vegetables ☐
**Quesadillas (tortilla with melted cheese)**	With corn tortilla ☐		With wheat flour tortilla ☐
**Tacos or burritos**	With corn tortilla ☐		With wheat flour tortilla ☐
**Soup**	Homemade with vegetables ☐		Instant soup ☐
**"Bolis", ice cream or ice pops**			Any presentation ☐
**Duros (corn chips)**			Any presentation ☐
**Pizza**			Any presentation ☐
**Molletes (bread rolls with cheese and ham)**			Any presentation ☐
**Pepihuates (coated peanuts and cucumber in a tomato based juice)**			Any presentation ☐
**Tamales**			Any presentation ☐
**Torta (bread roll filled with cheese, processed meats, vegetables)**			Any presentation ☐
**Others:**			

100% (or close to 100%) of the items available in the school canteen are from the 'green' or 'amber' classification ☐ YES ☐ NO

At least **50%** of the items available in the school canteen are from the 'green' or 'amber' classification ☐ YES ☐ NO

Table 4. Breakfast Menu Instrument

SCHOOL NUMBER: _________

Foods and beverages included in school breakfast menus

Foods and beverages using for preparation of food in school breakfast or lunch		
Green	Amber	Red
Vegetables ☐		
Whole grain cereals ☐		Non whole grain cereals ☐
Legumes and products of animal origin (eggs, meat, chicken etc.) ☐		Processed meats and sausages high in salt ☐
Fruits ☐		
Simple water ☐		
"Atoles" prepared with whole grains (oats, amaranth, barley, rice) ☐		"Atoles" prepared with NON whole grains (cornstarch, cookies, etc.) ☐
		Cream and butter ☐
Dried fruit ☐		
Nuts and seeds ☐		

Other foods added to the menu		
**Green**	**Amber**	**Red**
	Natural fruit juice (100% juice) without added sugar ☐	Natural fruit juice (100% juice) with added sugar ☐
		Processed juices and nectars ☐
		Processed juices and nectars with artificial sweeteners ☐
		Iced tea, sodas and other sugar sweetened beverages ☐
		Iced tea, sodas and other beverages with artificial sweetener ☐
Cheeses low in fat and/or salt ☐		Cheeses high in fat and/or salt ☐
Whole, low-fat or skim milk without added sugar ☐	Whole, low-fat or skim milk with artificial sweeteners ☐	Whole, low-fat or skim milk with added sugar ☐
Soy-based beverages without added sugar ☐	Soy-based beverages with artificial sweeteners ☐	Soy-based beverages with added sugar ☐
		Snacks (chips and other salty processed foods) ☐
		Cookies, cakes and other sweets ☐
Others: ☐	☐	☐

100% (or close to 100%) of the menu items are from the 'green' or 'amber' classification ➡ YES ☐ NO ☐

At least **50%** of the menu items are from the 'green' or 'amber' classification ➡ YES ☐ NO ☐

Table 5. Evaluation of the physical environment

SCHOOL NUMBER: _________

Structural Evaluation Based on Observation

Question	Answer		
**1.** Is there advertising directed at processed foods and/or beverages observed at school?	☐ No (0)	☐ Yes (1)	
**2.** Are there promoting material related with the Agreement in the school?	☐ No (0)	☐ Yes (1)	
**3.** Is there any mobile food vendor outside the school?<br>*Consider only those vendors on the same street in front of the school*	☐ No (0)	☐ Yes (1)<br>*If the answer is "no", continue with question 4. If the answer is "yes"…*	
Number of mobile food vendors outside the school:	____________________		
Category in which most part of the items sold by the mobile food vendors are:	☐ Red *SSBs and HSFAS* (0)	☐ Amber *Natural juice without sugar* (1)	☐ Green *Vegetables, whole grains* (2)
**4.** Are there water fountains or some kind of access to free drinking water in the school?	☐ No (0)	☐ Yes (1)<br>*If the answer is "no", skip the rest of the questions, if the answer is "yes"...*	
Specify the number of water fountains observed:	____________________		
Are all the water fountains functional?	☐ No (0)	☐ Yes (1)<br>*If the answer is "no", **specify how many are functional:** ______*	
Are all the water fountains clean?	☐ No (0)	☐ Yes (1)<br>*If the answer is "no", **specify how many are clean:** ______*	

Photographs:

2 Photographs from the outside (mobile food vendors)	☐ Yes	☐ No	Comments:
2 Photographs from the school canteen	☐ Yes	☐ No	
2 Photographs from food advertising (SSBs o HSFAS)	☐ Yes	☐ No	
2 Photographs from promotional material related with healthy habits	☐ Yes	☐ No	

Table 6. Non-participation Survey

SCHOOL NUMBER: _________

Instructions: In the case that the school authorities choose not to participate in the study, ask for the following information in a friendly manner.

NON-PARTICIPATION SURVEY

"Thank you for your time, before we leave we would like you to help us answer 3 quick questions, it would be very helpful"

1. Does the school have policies related to junk food or sugar sweetened beverages?

☐ No (0) ☐ Yes (1) ☐ I don't know (2)

2. Do you consider that your school has sufficient water fountains for the children that attend the school?

☐ No (0) ☐ Yes (1) ☐ I don't know (2)

3. In Mexico there is a guideline called "Agreement that establishes the general guidelines for the sale and distribution of prepared and processed foods and beverages in the schools of the National Education System." Have you received information about this Agreement?

☐ No (0) ☐ Yes (1)

Fig 1. Checklist

School number: ___________

CHECK LIST FOR THE VISIT

STEP 1 (Before the visit):

- ☐ Materials (7)
- ☐ Identification form (section 1)
- ☐ Assign the school's identification number
- ☐ Carry the project ID badge

STEP 2 (Arriving):

- ☐ Identification form (section 3)
- ☐ Enter the school (ask for the principal)
- ☐ Access denied

STEP 3 (Entrance):

- ☐ Introduce yourself
- ☐ Benefits of the project
- ☐ Informed consent
- ☐ Sign or Non participation survey
- ☐ Identification form (section 2)

STEP 4 (Interview):

- ☐ This is anonymous
- ☐ Explain the dynamics of the interview

STEP 5 (School menu):

- ☐ Menu instrument
- ☐ Ask for the person responsible for the menu
- ☐ If need to be contacted by email (write down email address)

STEP 6 (School canteen):

- ☐ Go to the school canteen
- ☐ Photos of the school canteen
- ☐ School Canteen instrument
- ☐ Is there any other place with food/beverages?

STEP 7 (Structural evaluation tool):

- ☐ Structural evaluation instrument
- ☐ Photos of food advertising or promotional material for the Agreement

STEP 8 (Goodbye):

- ☐ Say thank you
- ☐ A report will be sent
- ☐ Tools completed
- ☐ Leave
- ☐ Photos of mobile food vendors
