# Supplementary material for: Poor compliance with school food environment guidelines in elementary schools in Northwest Mexico: A cross-sectional study
Source: PLoS One. 2021 Nov 11;16(11):e0259720. doi: 10.1371/journal.pone.0259720 (PMC8584694; doi:10.1371/journal.pone.0259720)
Supplement: S1 Table — List of food and drinks and their prevalence in school canteens. (PDF) [file pone.0259720.s004.pdf]

S1 Table. Foods and beverages available in school canteens – List of food and drinks and their prevalence in school canteens.

Indicator	*Prevalence % (95% CI)*	
Green classification		
Bottled water	92.2	(85.3 - 96.6)
Fresh fruit and vegetables – whole or chopped	87.4	(79.4 - 93.1)
Whole grain cereals without added sugar (amaranth, outs or granola)	3.9	(1.1 - 9.6)
Seeds and nuts without added salt	1.9	(0.2-6.8)
Dried legumes without added salt (e.g. broad beans, broad beans)	1.9	(0.2-6.8)
Whole, low-fat or skim milk without added sugar	18.4	(11.5 - 27.3)
Milk-based beverages without added sugar (e.g. milkshakes, hot chocolate)	1.9	(0.2 - 6.8)
Soy drinks without added sugar	0	
Low fat and low salt cheeses	1.0	(0 - 5.3)
**Prepared foods**		
Whole grain sandwiches with vegetables	70.9	(61.1 - 79.4)
"Quesadilla" - corn tortilla with melted cheese	28.2	(19.7 - 37.9)
Tacos and "burritos" using a corn tortilla	20.4	(13.1 - 29.5)
Homemade soup with vegetables	26.2	(18.0 - 35.8)
Amber classification		
Natural fruit juices (100% juice) without added sugar	2.9	(0.6 - 8.3)
Whole or low-fat milk with artificial sweetener	0	
Milk-based beverages with artificial sweetener	1.0	(0 - 5.3)
Soy-based beverages with artificial sweetener	1.0	(0 - 5.3)
Red classification		
Natural fruit juices with added sugar	38.8	(29.4 - 48.9)
Whole grain cereals with added sugar	50.5	(40.5 - 60.5)
Seeds and nuts with added salt	7.8	(3.4 - 14.7)

Dried legumes with added salt (e.g. chickpeas, broad beans)	87.4	(79.4 - 93.1)
Milk with added sugar	12.6	(6.9 - 20.6)
Milk-based beverages with added sugar (e.g. chocolate milk, drinkable yogurt, milkshakes)	65.0	(55.0 - 74.2)
Sport or energy drinks	11.7	(6.2 - 19.5)
Soy-based beverages with added sugar	13.6	(7.6 - 21.8)
Cheeses high in fat and sodium	71.8	(62.1 - 80.3)
Processed juices and nectars	91.3	(84.1 - 95.9)
Iced tea, sodas and other sugar sweetened beverages	26.2	(18.0 - 35.8)
Snacks (potato chips and other salty processed foods)	72.8	(63.2 - 81.1)
Cookies, cakes, candies and sweets	98.1	(93.2 - 99.8)
**Prepared foods**		
White bread sandwiches without vegetables	38.8	(29.4 - 48.9)
"Quesadilla" – wheat flour tortilla with cheese	87.4	(79.4 - 93.1)
Tacos and "burritos" using wheat flour tortilla	91.3	(84.1 - 95.9)
Instant soup	1.0	(0 - 5.3)
Ice creams, ice pops and similar	83.5	(74.9 - 90.1)
Corn chips	26.2	(18.0 - 35.8)
Pizza and similar	33.0	(24.1 - 43.0)
"Molletes" – bread rolls with cheese and ham	72.8	(63.2 - 81.1)
"Pepihuates" – coated peanuts and cucumber in a tomato-based juice (includes salt, chili, sugar and lemon)	79.6	(70.5 - 86.9)
"Tamales" – a traditional dish made of masa (nixtamalized corn, lard) with a variety of fillings (e.g. meat or cheese), steamed in a corn husk	21.4	(13.9 - 30.5)
"Tortas" – bread rolls filled with a variety of meats, cheese, vegetables	76.7	(67.3 - 84.5)

CI – confidence interval

Note: data was collected by the data collectors through observation and classification of the items available in the school canteen. All visits were made from Monday to Thursday due to more liberal restrictions applying on Fridays.
