# Supplementary material for: Poor compliance with school food environment guidelines in elementary schools in Northwest Mexico: A cross-sectional study
Source: PLoS One. 2021 Nov 11;16(11):e0259720. doi: 10.1371/journal.pone.0259720 (PMC8584694; doi:10.1371/journal.pone.0259720)
Supplement: S2 Table — List of food and drinks and their prevalence in school breakfast menus. (PDF) [file pone.0259720.s005.pdf]

S2 Table. Foods and beverages included in school breakfast menus – List of food and drinks and their prevalence in school breakfast menus.

Indicator	Prevalence % (95% CI)	
Green classification		
Vegetables	46.4	(33.0-60.3)
Whole grain cereals	89.3	(78.1-96.0)
Legumes and products of animal origin (e.g. eggs, meat, chicken)	46.4	(33.0-60.3)
Fruits	50.0	(36.3-63.7)
Water	5.4	(1.1-14.9)
Atole (whole grain cereal-based hot beverage with milk)	14.3	(6.4-26.2)
Dried fruit	66.1	(52.2-78.2)
Nuts and seeds	67.9	(54.0-79.7)
Low fat and low salt cheese	7.1	(2.0-17.3)
Milk without added sugar	75.0	(61.6-85.6)
Soy-based beverages without added sugar	1.8	(0-9.6)
Amber classification		
Natural fruit juice (100% juice) without added sugar	1.8	(0-9.6)
Milk with added artificial sweetener	1.8	(0-9.6)
Soy-based beverages with artificial sweetener	0	
Red classification		
Non whole grain cereals	37.5	(24.9-51.5)
Processed meats and sausages high in salt	17.9	(8.9-30.4)
Cream and butter	5.4	(1.1-14.9)
Natural fruit juices with added sugar	8.9	(3.0-19.6)
Processed juices and nectars	1.8	(0-9.6)
Juices and nectars with added artificial sweetener	1.8	(0-9.6)
Iced tea, sodas and other sugar sweetened beverages	5.4	(1.1-14.9)
Iced tea, sodas and other beverages with artificial sweetener	0	

Cheeses high in fat and/or salt	28.6	(18.0-42.1)
Milk with added sugar	14.3	(6.4-26.2)
Soy-based beverages with added sugar	0	
Snacks (chips and other salty processed foods)	0	
Cookies, cakes and other sweets	5.4	(1.1-14.9)

CI – confidence interval

Note: data was collected by the data collectors through observation and classification of the items available on the breakfast menu (and conversation with the person responsible for the breakfast program to identify changes to the menu)
