# Supplementary material for: Poor compliance with school food environment guidelines in elementary schools in Northwest Mexico: A cross-sectional study
Source: PLoS One. 2021 Nov 11;16(11):e0259720. doi: 10.1371/journal.pone.0259720 (PMC8584694; doi:10.1371/journal.pone.0259720)
Supplement: Attachment — Submitted filename: Response to reviewers 19 Sept 2021.docx [file pone.0259720.s008.docx]

**Response to the reviewer**

1. The authors could report implementation and compliance rate by public schools and private schools, and by principal/teachers (administrative staff), to enrich their results. In doing so, the authors can help readers to further understand whether the implementation and compliance rates are different in public/private schools and etc.

Thank you for the suggestion. We investigated whether the implementation rate was higher in private schools than public schools and found that it was. Full implementation of the agreement was more likely to be reported by private schools than public schools (33.3 vs 9.0%, p=0.009, Fisher’s exact test). We have added this finding to the manuscript (lines 338-340). In relation to compliance, the total number of schools that are partially or fully complying with the agreement is too small (5/103) to enable a meaningful comparison between private and public schools. We are unsure what comparison the reviewer is alluding to in relation to principal/teachers (administrative staff).

2. The school authorities will always think that parents and students are barriers, while they are the facilitators to implantation of the AGGEEMENT. Therefore, figure 2 does not tell us new information. The authors should consider report the difference of reasons (such as lack of knowledge) between public and private schools. In doing so, the authors can help readers get new information about whether there are different reasons of poor compliance for different types of school.

Analysis of the reasons why the different groups/persons are barriers is complicated by the fact that the question reporting the reasons is not specific to each of the groups – thus the interpretation of any differences in the reasons is difficult. When we analyzed the differences between public and private schools regarding the reasons (without matching to the group/person), we only found a difference between public and private schools for ‘Lack of interest’. No differences were found for the other reasons. Thus, we have not added this information to the paper. Further, we think that the analysis/results shown in Table 3 is a more useful way to present this information. However, in response to the reviewer’s comment we have replaced figure 2 with a figure that compares the barriers and facilitators to implementation for public and private schools. The graph of barriers shows that parents, students and vendors were more likely to be reported as barriers for public schools than for private schools, which we have now noted in lines 380-382). And the graph of facilitators shows that parents were more likely to be reported as facilitators for private schools than for public schools (see lines 403-404).
