# Supplementary material for: Poor compliance with school food environment guidelines in elementary schools in Northwest Mexico: A cross-sectional study
Source: PLoS One. 2021 Nov 11;16(11):e0259720. doi: 10.1371/journal.pone.0259720 (PMC8584694; doi:10.1371/journal.pone.0259720)
Supplement: Attachment — Submitted filename: Hugues et al Response to reviewer comments.docx [file pone.0259720.s006.docx]

**Response to reviewer comments:**

Our responses follow each comment.

**Reviewer #1**: Referee report for Manuscript “PONE-D-20-39852”
This paper, entitled “Poor compliance with school food environment guidelines in elementary schools in Northwest Mexico: a cross-sectional study”, reports a survey result. The survey is conducted to collect data on the implementation of Federal Government guideline for the sale and distribution of food and beverage (AGREEMENT) in the context of high overweight and obese prevalence in Mexican. While the paper raises important questions, it has the following flaws:

1. it is a report, not a research article. It only reports the very low rate of compliance rates without analyzing the behind reasons. It could be extended, for example, by analyzing the spillover effect of mobile food vendors on the street on the provision of unhealthy (sugar-sweetened beverages) foods of public schools, or by examining the difference between public and private schools. Another way to extend it is to examine why government seems use secondary document, rather than the AGREEMENT, to inspect schools. Are there any corruptions?

**Response:**

We disagree with the reviewer on this aspect.  While the study is a descriptive cross-sectional study this is a valid epidemiological research design, and the study offers important and high-quality results (as noted by reviewer #2). This is the first study of a large, random sample of schools in Mexico, that has used direct observation of the school canteens and school yard to measure compliance rather than relying on self-reported data (which is more likely to lead to an overestimation of compliance). The observation method used followed a standardized protocol, is replicable and valid and therefore provides the opportunity to monitor progress. It was also a necessary first step to show that compliance was low and to highlight possible reasons for this, such as lack of training of the school authorities.

Further, while the objective of the study was not to analyze the reasons for low compliance, the discussion raises several potential explanations that may serve

as hypotheses for future studies.

2. The contribution of this paper to literature is quite limited. This paper brings us updated survey results, with findings similar to the previous literature.

Response:

We disagree that the findings are similar to the previous literature.  The findings from this study, using direct observation, are much lower than previous studies in Mexico that used self-report.  And, as mentioned in point 2, it will serve to show that there is indeed a problem with the implementation of the guidelines so that steps can be taken to improve both the guidelines themselves and their implementation. An additional strength of this type of study is that, the monitoring of food environments can help drive increased accountability of governments and the private sector and stimulate policy changes to improve them (Sacks et al. 2021). A comment has been added to the discussion (page 26) to this effect.

Sacks G, Kwon J, Vandevijvere S, Swinburn B. Benchmarking as a public health strategy for creating healthy food environments: an evaluation of the INFORMAS initiative (2012–2020). Annu Rev Public Health. 2021;42(1):345-62.

**Reviewer #2**: Using a random sample of schools in Hermosillo, Sonora, this study shows the poor compliance of 2014 AGREEMENT, which establishes the general guidelines for the sale and distribution of foods and beverages in schools in Mexico. Rather than relying on self-reported data, this study uses direct observation of the school canteens and school yard to measure compliance. These measures are performed to a high technical standard. The study further discusses the barriers and facilitators to implementation of the AGREEMENT in detail.

Response:

We thank you for your comments and agree that the use of direct observation was a strength of the study, as well as the high technical standards that we employed to ensure valid results.
