# Supplementary material for: Poor compliance with school food environment guidelines in elementary schools in Northwest Mexico: A cross-sectional study
Source: PLoS One. 2021 Nov 11;16(11):e0259720. doi: 10.1371/journal.pone.0259720 (PMC8584694; doi:10.1371/journal.pone.0259720)
Supplement: Attachment — Submitted filename: referee report-R1.docx [file pone.0259720.s007.docx]

The authors could report implementation and compliance rate by public schools and private schools, and by principal/teachers (administrative staff), to enrich their results. In doing so, the authors can help readers to further understand whether the implementation and compliance rates are different in public/private schools and etc.

The school authorities will always think that parents and students are barriers, while they are the facilitators to implantation of the AGGEEMENT. Therefore, figure 2 does not tell us new information. The authors should consider report the difference of reasons (such as lack of knowledge) between public and private schools. In doing so, the authors can help readers get new information about whether there are different reasons of poor compliance for different types of school.
